# SCINA: A Semi-Supervised Subtyping Algorithm of Single Cells and Bulk Samples 

**DOI:** 10.3390/genes10070531

**Published:** 2019-07-12

**Authors:** Ze Zhang, Danni Luo, Xue Zhong, Jin Huk Choi, Yuanqing Ma, Stacy Wang, Elena Mahrt, Wei Guo, Eric W Stawiski, Zora Modrusan, Somasekar Seshagiri, Payal Kapur, Gary C. Hon, James Brugarolas, Tao Wang

**Affiliations:** 1 Quantitative Biomedical Research Center, Department of Population and Data Sciences, University of Texas Southwestern Medical Center, Dallas, TX 75390, USA; 2 Bioinformatics Core Facility, University of Texas Southwestern Medical Center, Dallas, TX 75390, USA; 3 Center for the Genetics of Host Defense, University of Texas Southwestern Medical Center, Dallas, TX 75390, USA; 4 Kidney Cancer Program, Simmons Comprehensive Cancer Center, University of Texas Southwestern Medical Center, Dallas, TX 75390, USA; 5 Department of Internal Medicine, University of Texas Southwestern Medical Center, Dallas, Texas, TX 75390, USA; 6 BioHPC, University of Texas Southwestern Medical Center, Dallas, Texas, TX 75390, USA; 7 Molecular Biology Department, Genentech, Inc., South San Francisco, CA 94080, USA; 8 Bioinformatics and Computational Biology Department, Genentech, Inc., South San Francisco, CA 94080, USA; 9 Department of Pathology, University of Texas Southwestern Medical Center, Dallas, TX 75390, USA; 10 Laboratory of Regulatory Genomics, Cecil H. and Ida Green Center for Reproductive Biology Sciences, Division of Basic Reproductive Biology Research, Department of Obstetrics and Gynecology, University of Texas Southwestern Medical Center, Dallas, TX 75390, USA

**Keywords:** single-cell RNA-seq, CyTOF, SCINA, HLRCC, RCC, renal cell carcinoma, fumarase, fumarate hydratase

## Abstract

Advances in single-cell RNA sequencing (scRNA-Seq) have allowed for comprehensive analyses of single cell data. However, current analyses of scRNA-Seq data usually start from unsupervised clustering or visualization. These methods ignore prior knowledge of transcriptomes and the probable structures of the data. Moreover, cell identification heavily relies on subjective and possibly inaccurate human inspection afterwards. To address these analytical challenges, we developed SCINA (Semi-supervised Category Identification and Assignment), a semi-supervised model that exploits previously established gene signatures using an expectation–maximization (EM) algorithm. SCINA is applicable to scRNA-Seq and flow cytometry/CyTOF data, as well as other data of similar format. We applied SCINA to a wide range of datasets, and showed its accuracy, stability and efficiency, which exceeded most popular unsupervised approaches. SCINA discovered an intermediate stage of oligodendrocytes from mouse brain scRNA-Seq data. SCINA also detected immune cell population changes in cytometry data in a genetically-engineered mouse model. Furthermore, SCINA performed well with bulk gene expression data. Specifically, we identified a new kidney tumor clade with similarity to FH-deficient tumors (FHD), which we refer to as FHD-like tumors (FHDL). Overall, SCINA provides both methodological advances and biological insights from perspectives different from traditional analytical methods.

## 1. Introduction

Single cell profiling techniques such as single cell sequencing and cytometry are powerful tools for comprehensive and high-resolution characterization of cellular heterogeneities observed in tumors, brain, and other tissues. Single cell RNA-Seq (scRNA-Seq) measures the mRNA expression of several thousand genes from cells numbering a few hundred up to about 1 million, depending on the particular scRNA-Seq protocol, such as Smart-Seq [1] or the 10× Genomics Chromium [2]. Cytometry experiments such as FACS and the recent variation, CyTOF [3], can measure the expression of about 10–50 protein markers of up to 1 million cells. Many successful statistical methods, for example, Seurat [4], SINCERA [5], PhenoGraph [6] and SNN-Cliq [7] have been developed to identify cell types from these high-dimensional profiling data with dimension reduction algorithms, unsupervised clustering and visualization techniques.

**Figure 1:**
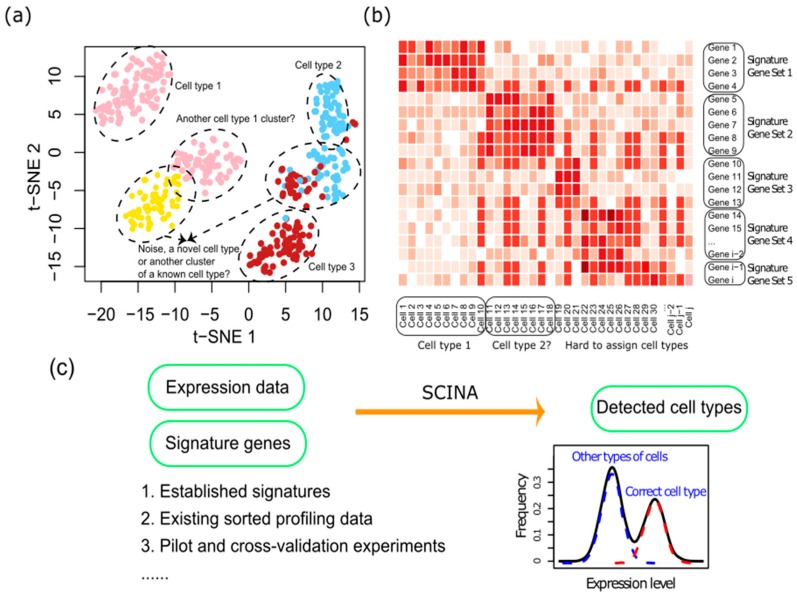
The Semi-supervised Category Identification and Assignment (SCINA) algorithm. (**a**) An illustrated t-SNE plot showing the problems often associated with discerning cell types based on clustering effect of t-SNE plots. (**b**) An illustrated heatmap showing the difficulties of manually assigning cell types based on signature genes when multiple signature genes are known for each cell type. (**c**) The rationale of SCINA. SCINA represent a supervised and automated approach for assigning cell types based on prior knowledge of signature genes and can directly arrive at detected cell types. The gene signatures could come from a variety of sources.

However, there are several major issues associated with unsupervised approaches. (i) Unsupervised algorithms such as K-means clustering (KC), t-SNE, etc., only cluster the cells into groups. These cell groups are then assigned to specific cell types based on human inspection of signature genes’ expression, which is often labor-intensive and subjective, especially on borderline cases (Figure 1a). For example, Rosenberg et al.’s SPLiT-seq paper manually merge 73 clusters into 9 cell types via visual examination of the expression of cell markers [8]. (ii) Furthermore, many cell types are identified by more than one gene (Figure 1b). For example, CD4 + T cells need to be identified by the expression of both CD3 and CD4. These signature genes need to be manually weighed when assigning cell types, leading to even more bias and obscurity. (iii) Thirdly, cell type clustering and assignment are split into two stages, where unsupervised cell clustering in the first stage of analysis ignores prior knowledge of major existing cell populations and their transcriptional features. This leads to suboptimal performance especially when new cell types and subtypes are present in the sequenced cell pool, as they cannot be readily differentiated in the results of unsupervised clustering. (iv) Lastly, in addition to studying one experimental condition, researchers often need to assess changes between conditions in terms of the population abundances of different types of cells. Such analyses are less amenable to unsupervised approaches, as the cell groups and cell types are defined ad hoc each time, without justification of the reproducibility of their definitions between conditions.

With the advancements made in high-throughput biomedical research, prior knowledge of cell types and their transcriptomic features has become widely available in many cases. For example, a number of cell type-specific signature sets have been published, such as Immunome [9] and eTME [10]. Large amounts of RNA-Seq datasets of sorted cell populations are also available from databases like the Expression Atlas (https://www.ebi.ac.uk/gxa/home), which could be analyzed to define gene signatures. Alternatively, signatures could also be flexibly defined from researchers’ own pilot or cross-validation experiments. These resources could be leveraged by an automated algorithm for both cluster detection and cell type assignment of single cell profiling data in a supervised manner (Figure 1c).

In this study, we developed the Semi-supervised Category Identification and Assignment (SCINA) algorithm. The pseudo-code of SCINA is shown in Figure S1. Implemented by an expectation-maximization model, SCINA leverages prior reference information and simultaneously performs cell type clustering and assignment for known cell types in a targeted manner. The prior knowledge includes a group of signature genes that are characteristically highly expressed in one type of cell, but not in other cell types. With the prior reference, SCINA searches for a segregation of the pool of profiled cells. Each subpopulation of cells highly expresses signature gene(s) specified by the researcher. The SCINA algorithm implements this by using a bi-modal distribution assumption of the expression of the signature genes. The subpopulation of cells that do not highly express the marker genes for any of the specified cell types will be designated as a novel cell type, whose exact identities can be determined in follow-up studies. SCINA is also general and can be applied to patient-level data for disease subtyping. Overall, SCINA addresses a critical research need for targeted cell type and subtype dissection in different scenarios of single cell profiling applications, which has been previously neglected.

## 2. Materials and Methods

### 2.1. The SCINA Algorithm

SCINA is regarded as semi-supervised because the prior knowledge of signature genes is built into the unsupervised estimation process. This is different from supervised learning, where the goal is to minimize a loss function to approximate the known labels of instances. But it is also different from un-supervised machine learning methods, as SCINA is carried out under a weak degree of supervision: which genes (signature genes) should be highly expressed in which types of cells is known. SCINA accepts a list of signature gene sets for a variety of cell types, and an expression matrix, which is assumed to have been pre-processed by the user with logarithmic transformation and/or any appropriate method of normalization if necessary. For each cell type, the signature can have one or more genes. The signature genes, by default, should be highly expressed in one particular cell type compared to all other cell types. Expression of genes that are characteristically lowly expressed in one cell type compared to the other cell types can be inverted so that the pseudo expression of this gene is high in that cell type. For example, let’s assume we have a group of cells with their cell types being (A, A, A, B), and their expression of a certain gene X being (5, 6, 4, 0). Let’s assume we know this gene X should be a low expression marker gene for cell type B. Therefore, SCINA will automatically calculate a pseudo expression of X, as low_X, which is (−5, −6, −4, 0). And the gene low_X is now a regular marker gene, whose high expression identifies the B cell type. The SCINA model assumes that there is a bimodal distribution for each signature gene, with the higher mode corresponding to the cell type(s), in which this gene is designated as a signature, and the lower mode corresponding to all the other cell types. In the pool of cells being analyzed by SCINA, the cells with overall low signature expression (all signatures) are designated as “unknown” (default mode). However, SCINA also implements a switch that turns off the searching of “unknown” cells. The technical details of SCINA are specified in File S1.

### 2.2. Simulation Data Generation and Adding Noise to the Prior Information

We simulated 30 lists of signature genes, with the size of each list being an integer drawn from a uniform distribution from 2 to 10. We assigned the number of cells to each simulated cell type, with the number generated according to the following weighting distributions: (1) For the 30 (R) cell types, we assigned weights

(1) $$W\left(w_{1},w_{2},\ldots ,w_{R}\right)$$

generated randomly from a uniform distribution from 1 to 50. (2) For the 0th signature (the ‘unknown’ cell type) we assigned the weight.

(2) $$w_{0}$$

generated randomly from a uniform distribution from 5 to 15. We generated a total of N cells according to this weighting distribution (N was either 4000 or 10,000 under different simulation conditions, details described in the Results section). For each gene, the gene expression in the set of cells with this gene being designated as a signature was simulated from a normal distribution, whose mean was drawn from a uniform distribution between 3 and 5, and standard deviation drawn from another uniform distribution between 10^−5^ and 3. For the expression of this gene in other cell types, the expression was drawn from a normal distribution with mean drawn from a uniform distribution between 10^−6^ and 0.001, and standard deviation drawn from a uniform distribution between 10^−5^ and 3. Expression of genes in the ‘unknown’ class of cells was also simulated from the second distribution.

Next we challenged the SCINA algorithm by adding noise to the signatures with four methods: (1) When constructing the expression matrix, only R-n signatures were really used, while the other n signatures were assigned with weights W as 0, thus they were not used and became “additional” gene signatures for non-existing cell types. R is 30 and n is from 1 to 29; (2) We simulated the expression matrix with all signatures but removed signatures of n cell types from the full list from the input of signature lists. The goal is to test whether these cell types will be correctly assigned as ‘unknown’ or incorrectly assigned to other known cell types. R is 30 and n is from 1 to 15, considering that researchers should have an overall understanding of the major cell types in their experiments; (3) For each of n cell types, we added noise genes randomly selected from non-signature genes into that signature to double the size of the signature. The noise genes were not used to generate expression data matrix, yet they were input as noise signature genes to obscure the valid signatures. We increased n from 1 to 29 in 29 tests in this scenario. (4) Simulation of dropouts. In the expression simulation of each signature gene for each cell, we randomly performed dropout (expression is simulated from the ‘non-signature gene’ mode with mean values close to 0) with a chance of n x 3.33% (n is from 1 to 10). The empirical drop-out rates of single cell RNA-Seq data are typically 5% to 15%.

### 2.3. Stk4 KO Mouse Related Experiments

The generation of *Stk4* KO mice and the flow cytometry experiments follow the protocols that we have described elsewhere [11]. For the Complete Blood Count (CBC) analysis, blood was collected into EDTA-coated MiniCollect blood collection tubes (Greiner Bio-One, Kremsmünster, Austria, catalog #VG-450474) via submandibular vein puncture from unanesthetized mice. 40 μL of blood from each mouse were aliquoted and analyzed by a Hemavet 950FS (Drew Scientific, Oxford, CT, USA). Using an electrical impedance and focused flow cell system, the Hemavet quantified the total number of white blood cells, eosinophils, neutrophils, basophils, and lymphocytes.

### 2.4. UTSW FHD/FHD-Like Patient Cohort

RNA-seq and exome-seq samples of RCC patients from our University of Texas Southwestern Medical Center (UTSW) Kidney Cancer Program (KCP) database including those mentioned in Durinck et al. [12] were pre-processed and analyzed. FH-deficient tumors (FHD) and FHD-like RCCs from 9 patients (XP108, XP515, UTSW85, UTSW86, UTSW87, XP878, XP552, UTSW88, and XP790) were reviewed by two expert genitourinary pathologists at UTSW (P.K.) and Memorial Sloan Kettering Cancer Center (V.R.). DNA and RNA were extracted and sequenced from their frozen tissue (5 patients) or FFPE blocks (4 patients) as available.

These studies were performed under a protocol approved by the Institutional Review Board (IRB) of the University of Texas Southwestern Medical Center that involves analyses of kidney cancer (or control) samples donated by patients. Approval numbers: 012011-190 and STU-22013-052. The studies were conducted in accordance with the Belmont Report and U.S. Common Rule guidelines. 

### 2.5. Genomics Analysis Pipelines

9 RNA-seq libraries for the 9 UTSW FHD/FHDL patient tumors (one replicate per patient) were prepared using the TruSeq RNA Sample Preparation kit (Illumina, San Diego, CA, USA). The libraries were multiplexed three per lane and sequenced on the HiSeq 2500 platform to obtain, on average, ~100 million paired-end (2 × 75-bp) reads per sample. Tophat2 with parameters “--num-threads 12 -g 10 --library-type fr-unstranded” was used to align the RNA-seq reads to the human genome (hg19). FeatureCounts [13] with parameters “-t exon -g gene_id -s 0 -T 12 --largestOverlap --minOverlap 3 -M --fraction --ignoreDup -p -P -B -C” was used to count gene expression levels.

Exome capture was performed using the Agilent SureSelect Human All Exon kit (50 Mb) (Agilent Technologies, Santa Clara, CA, USA). Exome capture libraries were sequenced on the HiSeq 2500 platform (Illumina) to generate 2 × 75-bp paired-end data. For each patient, we sequenced both the malignant tissue and also the normal tissue as control. Quality of exome-seq data was examined by NGS-QC-Toolkit [14]. Exome-seq reads were aligned to the hg19 genome by BWA-MEM [15]. Picard was used to add read group information and mark PCR duplicates. A GATK toolkit [16,17,18] was used to perform base quality score recalibration and local realignment around Indels. GATK HaplotypeCaller with SNP and Indel recalibration, MuTect [19], and VarScan [20] were used to call SNPs and Indels. Annovar was used to annotate SNPs and Indels [21]. Non-silent missense mutations predicted to be deleterious by either SIFT or Polyphen2 as well as other loss-of-function mutations were kept. Somatic mutations and germline mutations were annotated according to the mutation allele frequencies in the tumor and normal samples. Mutations that had a background mutation frequency > 1% in any of ExAC, Esp6500, 1000 Genome, HRC, or Kaviar were eliminated.

### 2.6. Statistical Analyses

All computations and statistical analyses were carried out in the R computing environment [22]. K-means clustering was carried out using the CellRanger software suite [2] (version 2.2.0). Reanalysis functions provided by the package, including run_pca and run_kmeans_clustering, were applied with default parameters, except for the enforcing of two clusters for the k-means clustering. For running t-SNE, we used the Rtsne R package (version 0.13). We set dims = 2 and theta = 1, and used default values for all other parameters. Seurat analysis was performed with the Seurat R toolkit (version 3.0.0), the functions and parameters setting followed the tutorial provided on the Seurat website [23]. SINCERA analysis was applied with the SINCERA R package (version 0.99.0), following the vignette from the Xu Lab [24]. PhenoGraph analysis was applied with the Rphenograph R package (version 0.99.1). The parameters were adjusted according to the dataset sizes to achieve the best performances. FACS/CyTOF data were downloaded or generated as fcs files and converted to numerical matrices with the R package cytofkit [25] (version 1.6.5). Gene ontology analyses were carried out using the GOrilla server [26,27]. Principal Component Analysis (PCA) of RCC gene expression data was conducted with scaling. All statistical tests are two-tailed. For all boxplots appearing in this study, box boundaries represent interquartile ranges, whiskers extend to the most extreme data point which is no more than 1.5 times the interquartile range, and the line in the middle of the box represents the median.

### 2.7. Availability of Data and Material

UTSW RCC patients were asked if they would specifically consent to placement of their raw genomic data in a protected publicly accessible database. The RNA-Seq and exome-seq data of 5 consented UTSW FHD/FHD-like patients can be downloaded from the European Genome-phenome Archive with accession number EGAS00001002646 through controlled access.

## 3. Results

### 3.1. Validation of the SCINA Model by Simulation Data

**Figure 2:**
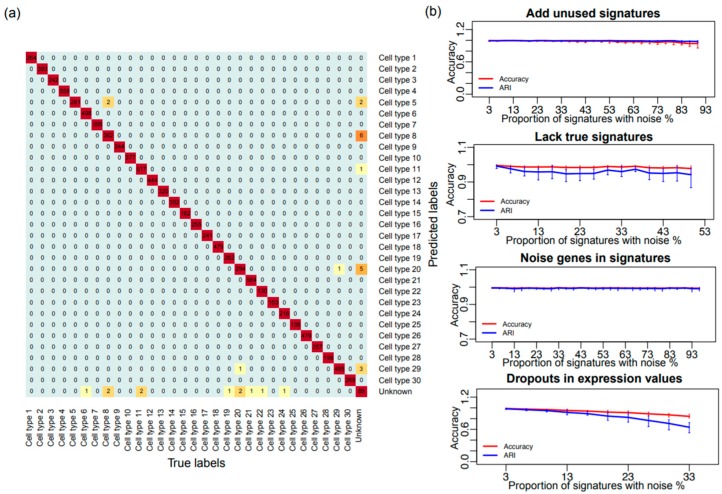
Performance of SCINA on simulated data. (**a**) Heatmap showing the overlap between the simulated cell types and the detected cell types by SCINA. (**b**) Challenging the SCINA algorithm by adding different types of noise: additional signatures for non-existent cell types, lack of true signatures for existing cell types, extra irrelevant genes in signatures, and simulated gene expression dropouts. Each scenario was repeated 10 times, and each repeat was performed on a newly-simulated matrix of 4000 cells. Performance is judged by Adjusted Random Index (ARI) and percentage of cells correctly assigned (ACC).

We first created a simulation dataset, by simulating 10,000 cells of 30 cell types and one additional subset of cells as the novel cell type (Figure 2a). On this dataset, SCINA yielded a classification accuracy (ACC) of 99.70%. Adjusted random index (ARI) is another metric for scoring the accuracy of categorical classifications [28], with 100% representing perfect agreement and ~0% representing random guess. Our result showed a 99.57% ARI between the true labels and the SCINA classifications. On the same dataset, K-means clustering yielded an ACC of 54.60%, and an ARI of 59.25% (Figure S2a), and Seurat provided an ACC of 82.30%, and an ARI of 62.13% (Figure S2b). SINCERA provided an ACC of 72.90%, an ARI of 57.74%, and for PhenoGraph the ACC was 84.93% and the ARI was 69.97%. We created variations of the simulation settings, where high ACCs and ARIs for SCINA were consistently observed (Table S1). As SCINA highly depends on prior information, we carefully investigated the sensitivity on the prior information by adding noise with four methods including: (1) “additional” gene signatures for non-existing cell types (Figure 2b, top panel, and Figure S4a,e); (2) simulated expression matrix with all signatures but removal of a certain number of signatures from the input signature gene lists (Figure 2b, upper middle panel, and Figure S4b,f); (3) ‘noise’ genes randomly selected from non-signature genes into each signature (Figure 2b, lower middle panel, and Figure S4c,g). (4) dropouts of reads in expression values of signature genes (Figure 2b, bottom panel, and Figure S4d,h). Details of generation of noise were described in the method section. To our satisfaction, the ACC and ARI of SCINA remained reasonably stable across all disturbances. In particular for dropout, as SCINA relies on signature genes that are abundantly expressed, the real dropout rate is likely to be much smaller than the range covered by this simulation. To further increase the difficulty of the simulation, we carried out more analyses where we added two or three of these types of noises simultaneously to the signatures, with the range of the total ‘obscured’ signature proportions being from 0% to 50% (Figure S3). We found that even in these complicated simulation scenarios, SCINA still achieved accurate performance. To compare the performance between SCINA and other benchmarking algorithms, we applied K-means clustering (KC), Seurat, SINCERA and PhenoGraph to the simulated datasets with the same noise in Figure 2b. The performance of SCINA and benchmarking software all decreased with the increased proportion of noise (Figure S4). However, within all the noise challenges, the performance of SCINA was better and more stable than the performance of other software.

We also created synthetic data based on combinations of real data, and evaluated the performance of SCINA. For this purpose, two clean populations of cells, Jurkat T cells and HEK293 [2] were mixed. Independent expression data of these two cell lines from Dilworth et al. [29] and GSE69511 were used to define signature genes. The mixing ratios ranged from 1:99 to 99:1, with a total of 2000 cells in each simulation. During cell type assignment, SCINA demonstrated accurate performance with near-perfect ARI Figure S5a) and ACC (Figure S5b) across all ranges of mixing ratios. In contrast, K-means clustering (KC), Seurat, SINCERA and PhenoGraph yielded unstable performance on these data, especially with unbalanced mixing ratios. Furthermore, we repeatedly simulated expression data 100 times with the same approaches as Figure 2a and calculated the probability of each cell being correctly assigned to its true cell type. The average correct assignment probability of the total of 4000 cells was 99.57% for SCINA, 54.31% for KC, 72.90% for SINCERA and 15.11% for PhenoGraph (Figure S5c, P < 10^−5^), which indicated that SCINA achieved a much more stable performance than unsupervised clustering. Most importantly, all the unsupervised approaches we applied could not assign the clusters of cells to the exact cell types, which was done manually by us.

### 3.2. Validation of the SCINA Model by Real Data

**Figure 3:**
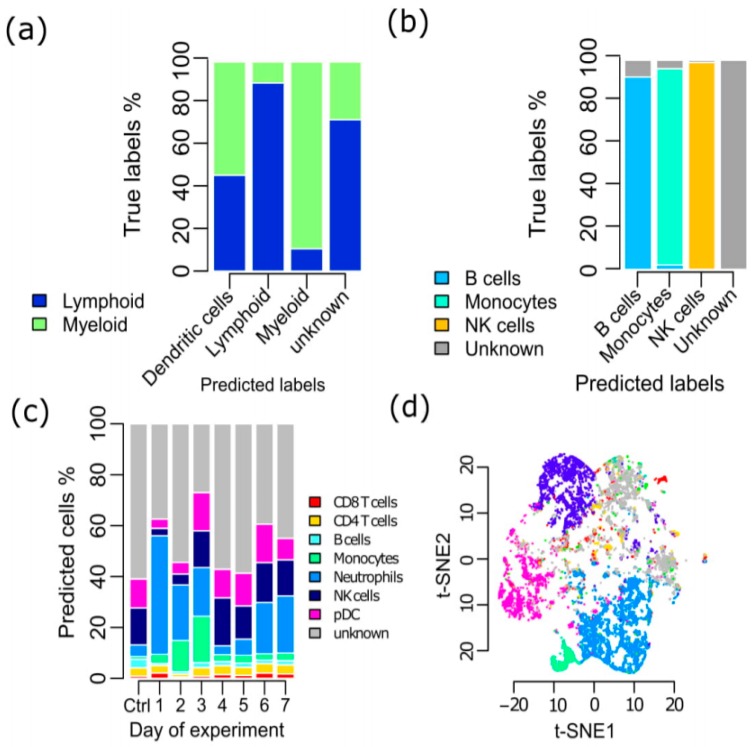
Validation of SCINA on real data (**a**) SCINA identified the cell types of CD45+ single cells enriched from Renal Cell Carcinomas (RCCs). Dendritic cells were left out of this analysis, as they could be of either lymphoid or myeloid lineage. (**b**) SCINA identified the cell types in a pool of cells comprised of B cells, monocytes, NK cells, and a “pseudo” unknown cell type. (**c**) SCINA was used to analyze the mouse CyTOF data collected each day following gland injury, which profiled an average of 389,777 cells at each time point. (**d**) t-SNE was used to analyze the same mouse CyTOF dataset. The cells were colored by cell types assigned by SCINA.

We applied SCINA to a pool of 1155 FACS-sorted CD45+ cells extracted from 6 clear RCC tumors (Figure S6). We employed our eTME gene signatures [10] for SCINA, which are highly specific for immune cells in RCCs. In this single cell experiment, the lymphoid and myeloid cells were enriched and sequenced separately, so that the lymphoid/myeloid identity of the cells was known. We scored whether each type of detected immune cells indeed belonged to the correct sub-pool of lymphoid and myeloid cells. Dendritic cells were left out of this analysis, as they could be of either lymphoid or myeloid lineage [30]. Overall, there was an accuracy (ACC) rate of 89.68% (Figure 3a). In sharp contrast, KC yielded two clusters of cells with poor concordance with the true lymphoid/myeloid labels (ACC of 64.50%). The more advanced unsupervised methods had a moderate better performance, with Seurat yielded and ACC of 60.10%, SINCERA of 81.03% and PhenoGraph of 69.00%. Furthermore, we conjectured that if at the pilot stage of one single cell project, the different types of cells could be sorted and sequenced to define de novo signatures, an even higher accuracy in subsequent experiments could be achieved. To mimic this process, we sampled 500 cells from the B cell, monocyte, and NK cell pools respectively from Zheng et al. and defined a set of de novo gene signatures [2]. Then the rest of these types of cells (n = 29,345) from the same study were mixed, along with CD4 T helper cells as the pseudo “unknown” cell type. As we expected, SCINA achieved a 97.3% ACC on this dataset (Figure 3b). Meanwhile, KC yielded an ACC of only 50.2%, Seurat provided a prediction ACC of 64.44%. The mixed dataset exceeded the optimized data scale of SINCERA and PhenoGraph, so we randomly select a subset containing 10% of the cells. The ACC performance of SINCERA and PhenoGraph on the subset was 54.44%, and 56.26%. Next, we downloaded the CyTOF data from Hawley et al. [31]. In this study, the mouse lacrimal gland was acutely injured via intraglandular injection of IL-1α, and the lacrimal gland single cell suspensions from day 1 to day 7 following injury were collected and subjected to CyTOF analysis. SCINA was applied on this dataset with established protein surface markers and discovered that neutrophil contents peaked at day 1 following injury and monocyte contents peaked at days 2 and 3 (Figure 3c), which is consistent with the overall conclusion of the original publication. In comparison, t-SNE was applied to cluster and visualize the single cells. The t-SNE plot (Figure 3d) shows that cells of the same type, assigned by SCINA, indeed cluster together, confirming the accurate performance of SCINA. However, there is a lack of clear boundaries between different types of cells on the t-SNE plane, making it impossible to use t-SNE as the sole methodology for assigning cell types in CyTOF data.

### 3.3. Discovery of a New Stage of Oligodendrocyte Development in Mouse Brain

**Figure 4:**
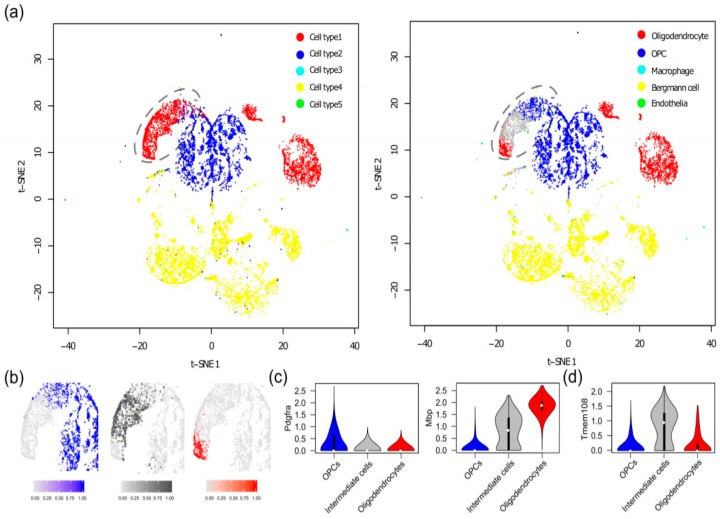
Discovery of a new stage of oligodendrocyte development in mouse brain. (**a**) t-SNE plot showing the clusters of cells detected by the manual inspection method employed in the original publication (left) and the cell types assigned by SCINA (right). (**b**) Density plots of cell type assignment probabilities generated by SCINA. (**c**) Violin plots showing the expression of *Mbp* and *Pdgfra* and (**d**) violin plot showing the expression of *Tmem108* in oligodendrocyte precursor cells (OPCs), oligodendrocytes, and the intermediate stage of cells.

To demonstrate the capability of SCINA to derive novel biological discoveries, we applied SCINA on the mouse brain scRNA-Seq data from Rosenberg et al. [8]. The original publication performed a cell type assignment based on painstaking manual inspection of unsupervised clustering results. In contrast, SCINA automatically generated cell type predictions for the 27,096 non-neuronal cells with the same markers used by Rosenberg et al., but expanded to also include the most positively correlated genes (as the original publication only identified one single gene as the marker for each cell type, which is too few for SCINA to operate). Overall, the SCINA-assigned cell types for most cells were consistent with those by the manual inspection method (Figure 4a). However, the manual method leads to the intertwined boundaries of the oligodendrocytes and oligodendrocyte precursor cells (OPCs) (the circles in Figure 4a). In comparison, SCINA divided the cells in this region into oligodendrocytes, OPCs and an unknown cell type. The density plots of cell type assignment probabilities (Figure 4b) demonstrate confident and clear separations between these three types of cells. We examined the expression of *Mbp*, marker for oligodendrocytes; and *Pdgfra*, marker for OPCs, which were used by Rosenberg et al. The findings also supported the grouping of these cells into an independent type (Figure 4c). In fact, these cells indeed seem to possess a unique transcriptomic program. We identified genes that were up-regulated in the unknown cells but down-regulated in both oligodendrocytes and OPCs. One of the top genes was *Tmem108*, which has been previously linked with schizophrenia and alcoholism [32,33] (*p*-values < 10^−5^ for comparing these cells with OPCs and oligodendrocytes, Figure 4d). Gene ontology analysis also confirmed the existence of differentially enriched pathways related to cell projection regulation in these cells (Table S2). Overall, the unknown cell type marked by SCINA could likely represent a newly-defined intermediate stage between OPC and oligodendrocyte development in mouse.

### 3.4. SCINA Detected Immune Cell Population Alterations in Stk4 Knock-Out Mice

**Figure 5:**
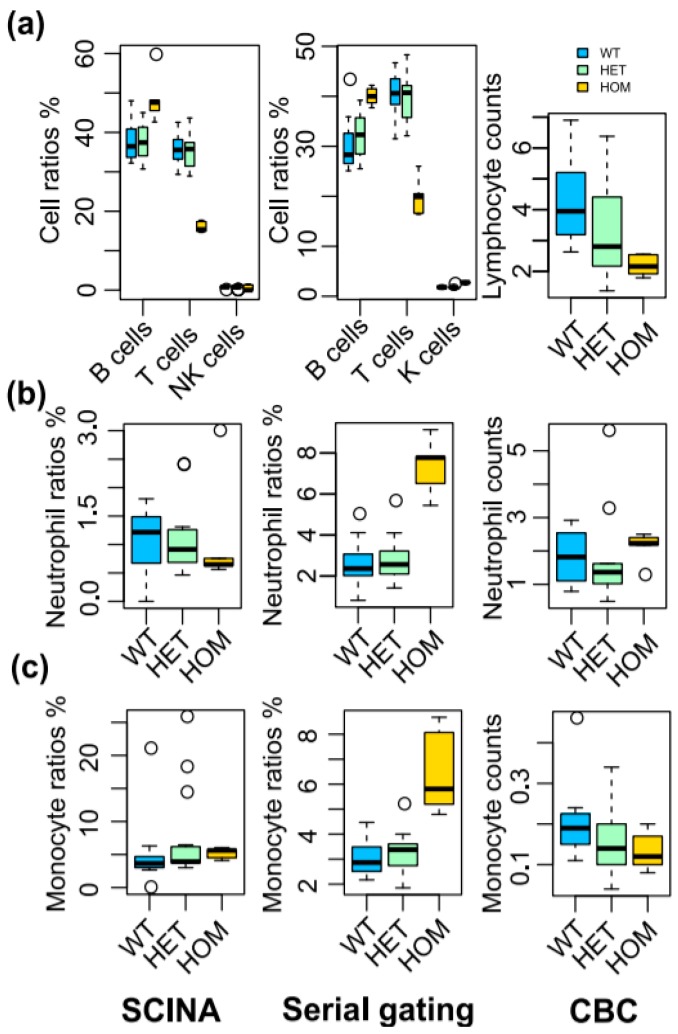
SCINA detects immune cell alterations in Stk4 KO mice. The pedigree contained a total of 12 wild-type (WT) mice, 15 heterozygous (HET) mice, and 5 homozygous (HOM) mice. The relative levels of immune cell populations are detected by SCINA from FACS data (left), the serial gating method from FACS data (middle), and CBC (right). (**a**) Lymphocytes, (**b**) Neutrophils, and (**c**) Macrophages.

Next, we evaluated SCINA on cytometry data. We generated a pedigree of CRISPR knock-out (KO) mice in *Stk4*, which is a key regulator of the Hippo pathway [34]. The blood sample of each mouse was run through a standard FACS pipeline as previously reported [11]. We had established a manual serial gating schema to sort and identify the populations of each type of immune cell from the FACS data using FlowJo (https://www.flowjo.com/). These FACS data were also analyzed by SCINA based on commonly used cell surface markers. We investigated the abundances of T cells, B cells, NK cells, macrophages, and neutrophils from all mice in the context of the *Stk4* genotype. Both the SCINA and manual gating methods detected lower T cell levels (P < 10^−5^ for both SCINA and serial gating), and elevated B cell levels (P = 3.93 × 10^−4^ for SCINA, P = 1.87 × 10^−3^ for serial gating) and NK cell levels (P = 0.008 for SCINA, P < 10^−5^ for serial gating) (Figure 5a). The T cell phenotype is consistent with previous reports in both human and mouse models that observed a correlation between *Stk4* deficiency and a paucity in T cells [35,36]. However, the manual gating method concluded that *Stk4* KO led to a significant increase in both neutrophils (Figure 5b, P < 10^−5^) and monocytes (Figure 5c, P < 10^−5^), while the neutrophil and monocyte counts are not significantly different between wild type and KO mice according to SCINA (P = 0.976 and P = 0.577). To resolve this conflict, we carried out a Complete Blood Count (CBC) analysis using blood from this pedigree of mice. We confirmed the insignificant changes of neutrophil and monocyte counts after knocking out *Stk4* (P = 0.496 for neutrophils and P = 0.209 for monocytes), which were in concordance with the SCINA results. Overall, these experiments demonstrated the ability of SCINA to yield biological discoveries while pruning spurious errors of manual cytometry analytical methods.

### 3.5. SCINA Identified a Novel Tumor Clade Based on Patient Bulk RNA-Seq Profiles

SCINA also solves a general supervised classification problem, for example, for patient-level sequencing data. Renal cell carcinomas (RCCs) are mainly comprised of clear cell RCC (ccRCC), papillary RCC (pRCC), and chromophobe RCC (chRCC). One minor but aggressive subtype of RCC has been recently identified: hereditary leiomyomatosis and renal cell cancer (HLRCC), which bears morphological similarities to high-grade pRCC and is characterized by germline mutations of the Fumarate Hydratase (*FH*) gene [37]. We collected TCGA pan-RCC RNA-Seq data from the Broad GDAC Firehose (n = 922) and also from the Kidney Cancer Program (KCP) at UT Southwestern Medical Center (n = 146) [12]. Preliminary pathological reviews determined that these RCCs were comprised of 528 ccRCCs, 323 pRCCs, 45 chRCCs, and 11 *FH*-deficient (FHD) samples (9 with germline mutations and 2 with somatic mutations in *FH*), along with 163 adjacent normal kidney samples (Table 1). Assuming most of these pathological classifications were correct, we defined gene signatures for each tumor type and for the normal kidney tissue based on differential expression analyses. The selected gene signatures consist of the top 20 genes most up-regulated in each tissue type (Table S3). SCINA was then applied using these gene signatures to the transcriptomic data to re-classify the patient subtypes, which resulted in a 91.9% concordance rate when comparing with the original pathological review (Table 1). However, there were 16 TCGA ccRCCs by pathological standards that were reclassified as chRCCs by SCINA. Interestingly, a recent second pathological review of the TCGA cohort suggested that 11 of these 16 RCCs could indeed be chRCCs [38].

**Figure 6:**
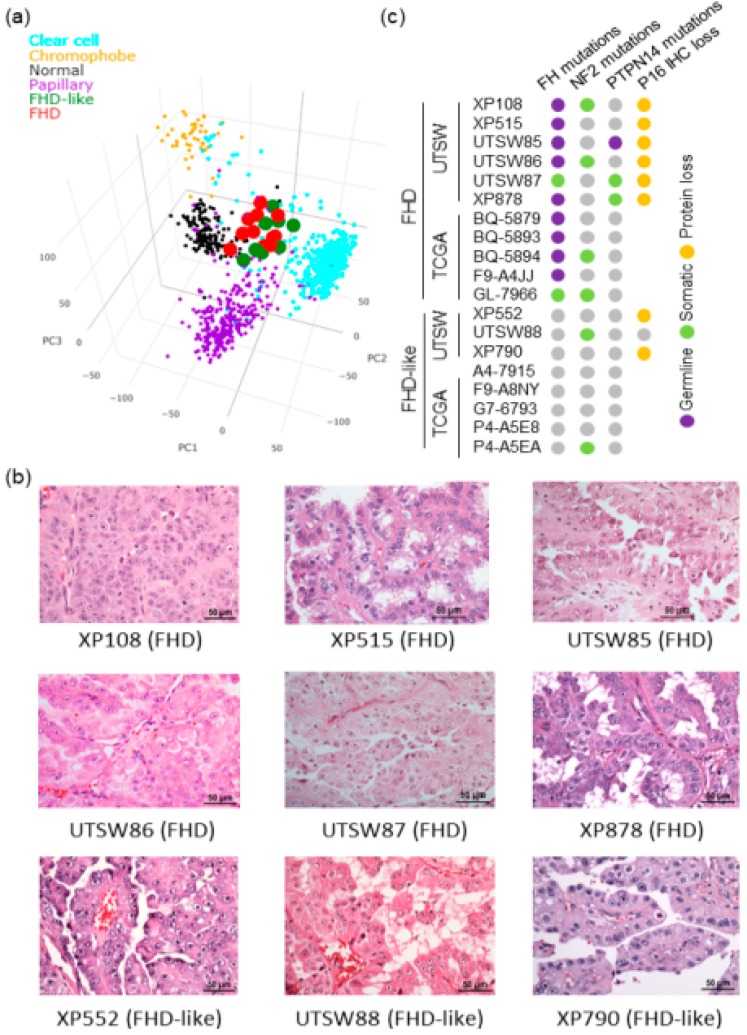
SCINA identified a novel RCC tumor clade based on gene expression profiling. (**a**) 3D Principal Component Analysis (PCA) plot showing clustering of UTSW KCP and TCGA RCC and normal kidney samples by expression (n = 1068). (**b**) H&E stained sections showing that both FH-deficient (FHD) and FHD-like tumors had features characteristic of hereditary leiomyomatosis and renal cell cancer (HLRCC), including papillary architecture, large nuclei, and prominent eosinophilic macronucleoli, with perinucleolar clearing. (**c**) Somatic/germline mutations and IHC results for FHD and FHD-like tumors.

Most interestingly, SCINA classified 8 pRCCs tumors as FHDs, which inspired us to carry out in-depth analyses to resolve this conflict. First, PCA based on the whole transcriptome (Figure 6a) confirmed that these 8 tumors clustered closely with the 11 FHDs. Pathological review by a GU pathologist (PK) revealed morphology features indistinguishable between FHDs and these 8 tumors (Figure 6b). However, these 8 tumors did not have any *FH* mutations and are not FHD tumors by definition (Figure 6c). Furthermore, for 3 tumors, where samples were available (UTSW cohort), immunohistochemistry showed preserved FH protein expression. We speculated that these tumors could represent a closely related tumor clade discovered by SCINA analysis of RNA-Seq data. We named them “FHD-like” and examined whether FHD and FHD-like tumors share convergent molecular perturbations. Interestingly, both FHDs and FHD-like tumors were enriched for mutations in *NF2*, which is in the Hippo pathway, and had frequent loss of p16 expression (UTSW cohort) (Figure 6c). Furthermore, the TCGA analysis demonstrated hypermethylation of *CDKN2A* (p16) in the 5 FHD-like tumors [39]. Overall, these analyses attest to the capability of SCINAto discover new tumor subtypes.

### 3.6. The SCINA R Package and SCINA on the Cloud

**Figure 7:**
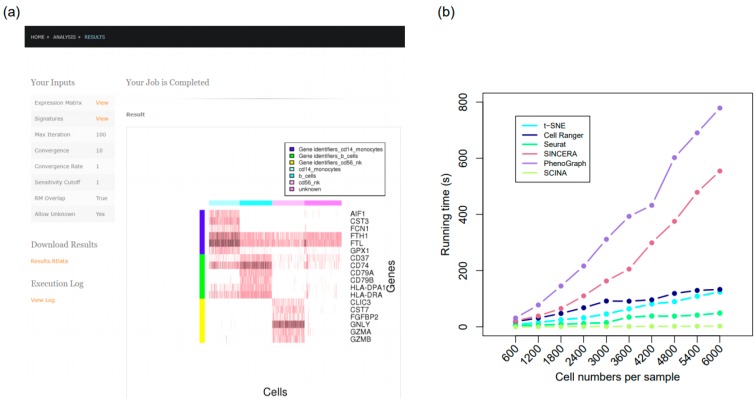
The SCINA R package and web server. (**a**) Screenshot of the SCINA web server. (**b**) Runtime comparison for SCINA, Cell Ranger (K-means clustering mode with graph-based fine-tuning), t-SNE, Seurat, SINCERA, and PhenoGraph. Runtime tests were performed on 10 datasets, constructed with 280n randomly selected HEK293 cells (out of 2885 total cells) and 320n randomly selected Jurkat cells (out of 3258 total cells), where n was an integer ranging from 1 to 10. Both HEK293 cell and Jurkat cell sets were publicly available from Zheng et al. [2].

For convenience of biologists and bioinformaticians who wish to apply SCINA in their research, we have created an R package [40,41] and also a web server (Figure 7a, [42]) to host SCINA on the cloud. Importantly, the R package/webserver has provided a functionality (the heatmap) for users to visually assess the degree of bi-modal distribution of each signature gene in the particular application. SCINA is extremely computationally efficient. On the same datasets, SCINA is 20 times to over 100 times faster than Seurat, t-SNE, and Cell Ranger, and 200 to 400 times faster than SINCERA and PhenoGraph (Figure 7b).

## 4. Discussion

SCINA is a novel methodology for cell type classifications in scRNA-Seq, CyTOF/FACS data, or even bulk RNA-Seq. Compared with unsupervised approaches, SCINA accepts the signatures of any number of marker genes, and weighing of the genes for each cell type is determined automatically by the algorithm. It is also generally applicable as a classification algorithm when data of similar formats are available. In our study, the performance of SCINA was comprehensively validated on a variety of datasets, which showed the accurate performance of SCINA and its superiority to other unsupervised software packages. This superiority is likely to be more pronounced as complexity increases, such as with the difficult problem of dealing with 30 cell types in Figure 2 and the unbalanced mixing proportions in Figure S5a,b. Nevertheless, the performance of SCINA is slightly influenced by the size of datasets, the total numbers of the cell types in the datasets, and the signature gene numbers for every cell type. As shown in Table S1 and in Figures S3–S4, incomplete signature genes for any cell types could decrease the performance of SCINA, so inclusion of more signature genes, in addition to the most well-established ones, is encouraged for more stable performance. Despite being built upon the bi-modality assumption, SCINA does not necessarily require each gene to have a clear and wide separation into two expression ranges. Some signature genes may have gradual changes along a differential or functional trajectory. Instead, SCINA relies on the separation of clusters of cells in a high-dimensional space formed by the contribution from all the signature genes. Therefore, SCINA requires less manual intervention and is more robust and objective.

Nevertheless, SCINA is synergistic and complementary to the other unsupervised methods. One project may start by applying unsupervised methods like t-SNE and ad hoc analyses for visualization to identify new cell types or subtypes. Researchers may then use SCINA and the de novo signatures to validate and refine their findings to detect these newly defined cell populations in subsequent experiments and understand how they change with different perturbations.

Prior knowledge of signature genes is available to the researchers in many biomedical research settings, which could come from several sources: (i) previously published signatures, (ii) pre-existing sequencing data of sorted cells, and (iii) pilot or cross-validation experiments where de novo signatures are defined. The third option is also a completely novel but very useful application of SCINA that cannot be afforded by unsupervised methods, which allows the researchers to assess the reproducibility of their biological phenomenon of interest across experimental conditions and replicates. As researchers will only need to apply signatures identified in one condition to another, rather than directly compare the expression data, batch effects are minimized. On the other hand, SCINA could also be regarded as a discovery tool for novel cell type-specific signatures. For example, cell types in the single cell profiling data can be defined using a smaller pre-defined set, based on which one may define additional marker genes. This process can be conducted iteratively and monitored by other analyses to verify the biological significance of the findings. Finally, when signature genes of multiple sources are available, the user has the flexibility of either merging some of these signatures, or choosing one of them for carrying out the SCINA analysis. One of the strengths of SCINA is that it provides users with flexibility to choose the optimal signatures, based on experimenting with different approaches to signature selection on the real data. To help with this process, we provide users with a function named plot.heat SCINA in the R package (and on the webserver) to generate a gene expression heatmap of signature genes for visual assessment.

One limitation of our approach is that it only considers genes which are signature genes, without considering the fact that some genes are stronger markers of a cell type. This limitation could be addressed by specifying a particular variance structure in the estimation process of SCINA to enforce differential weighting of genes. 

Overall, SCINA, the first semi-supervised “signature-to-category” cell type classification algorithm for single cell profiling data, addresses a critical research need that has been previously neglected. When coupled with other “category-to-signature” methods, SCINA could greatly enhance the flexibility and power of single cell profiling to propel biological discoveries. With more reference single cell datasets, such as those from the Tabula Muris project, supervised analyses of single cells, such as SCINA, will quickly become more feasible, useful and relevant.

## Figures and Tables

**Table 1:** Overlapping RCC subtypes assigned by pathological reviews and subtypes assigned by SCINA.

		SCINA-predicted Tumor Subtypes						
		Chromophobe	Clear Cell	FHD	Normal	Papillary	Unknown	Sum
**Pathologic review**	**Chromophobe**	44	0	0	1	0	0	45
	**Clear cell**	**16**	492	0	3	7	10	528
	**FHD**	0	0	11	0	0	0	11
	**Normal**	0	1	1	156	2	1	161
	**Papillary**	3	**8**	8	3	278	23	323
	**Sum**	63	501	20	163	287	34	1068

## Supplementary Materials

The following are available online at https://www.mdpi.com/2073-4425/10/7/531/s1, **Figure S1**, The pseudo-code of SCINA algorithm. **Figure S2**, Comparison of SCINA and other algorithms on simulation data. KC (a) and Seurat (b) analyses were performed on the same simulated dataset as in Figure S2a. The heatmap showed the overlap between the simulated cell types and the detected cell types by KC/Seurat. Each cluster detected by KC/Seurat was assigned to the simulated cell type with the largest overlap. **Figure S3** Combination of two (a) or three (b) types of noises to the signature genes in the simulation data. Noise type 1 to 4 corresponded to the 4 noise types in Figure S2b and details were described in the method section. For each combination, the proportions of signatures with denoted noise were increased from 0% to 50%. Different types of noises were added to signatures randomly and evenly. **Figure S4**, SCINA, KC, Seurat, SINCERA and PhenoGraph were applied to simulated datasets with different types of noise. The noise types were the same as in Figure S2b. Each scenario was repeated for 10 times, and each repeat was performed on a newly-simulated matrix of 4000 cells. Performance is judged by percentage of cells correctly assigned (ACC) (a–d) and by Adjusted Random Index (ARI) (e–h). The average ACCs or ARIs of 10 repeats were plotted. **Figure S5**, SCINA, KC, Seurat, SINCERA and PhenoGraph were used to identify Jurkat T cells and HEK293 cells. Accuracy is measured by Adjusted Random Index (ARI) (a) and the percentage of cells correctly assigned (ACC) (b). (c) Violin plot showing probabilities of all simulated cells being assigned to their ‘true’ cell types. Analysis were performed on 100 datasets, each with 4000 cells simulated with the same approaches as in Figure S2a. **Figure S6**, Analysis of the CD45+ single cells enriched from the RCC tumor microenvironment. (a) SCINA identified cell types based on eTME signatures containing signature genes of 25 immune cell types. Performance is measured by percentage of cells correctly assigned (ACC). Bars are colored differently according to the gold standard of the lymphoid and myeloid pool labels. (b–e) K-means, Seurat, SINCERA and PhenoGraph coupled with manual assignment of detected clusters to the lymphoid/myeloid cell pools. **File S1**, Full technical details of the SCINA model. **Table S1**, Accuracy of SCINA in simulated datasets of varying parameter settings. These datasets are all variants of the simulation dataset in Fig. 2a, but have different number of genes, cell types, cells, and signature genes for each cell type. **Table S2**, Enriched Biological Process ontology terms in OPCs, oligodendrocytes, and the intermediate stage of cells. The top 10 Gene Ontology terms are shown for each cell type. **Table S3**, Gene signatures used for the RCC bulk tumor analysis.
