# Supplementary material for: SCINA: A Semi-Supervised Subtyping Algorithm of Single Cells and Bulk Samples
Source: Genes (Basel). 2019 Jul 12;10(7):531. doi: 10.3390/genes10070531 (PMC6678337; doi:10.3390/genes10070531)
Supplement: Supplementary file 1 [file genes-10-00531-s001.zip › Supplementary Materials/Figure S1.pdf]

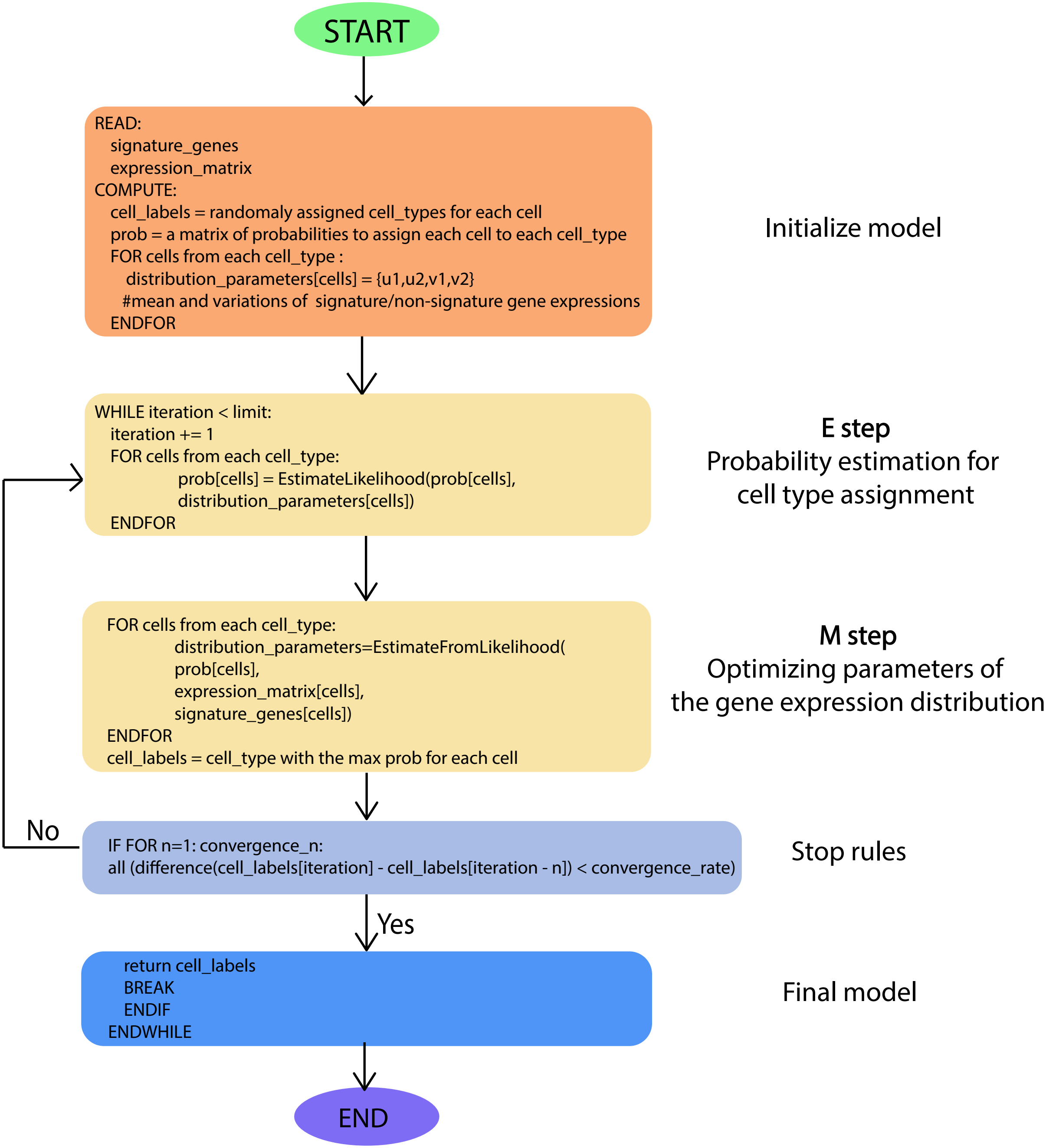
START
READ:
signature_genes
expression_matrix
COMPUTE:
cell_labels = randomaly assigned cell_types for each cell
prob = a matrix of probabilities to assign each cell to each cell_type
FOR cells from each cell_type :
distribution_parameters[cells] = {u1,u2,v1,v2}
#mean and variations of signature/non-signature gene expressions
ENDFOR
Initialize model
WHILE iteration < limit:
iteration += 1
FOR cells from each cell_type:
prob[cells] = EstimateLikelihood(prob[cells],
distribution_parameters[cells])
ENDFOR
E step
Probability estimation for
cell type assignment
FOR cells from each cell_type:
distribution_parameters=EstimateFromLikelihood(
prob[cells],
expression_matrix[cells],
signature_genes[cells])
ENDFOR
cell_labels = cell_type with the max prob for each cell
M step
Optimizing parameters of
the gene expression distribution
No
IF FOR n=1: convergence_n:
all (difference(cell_labels[iteration] - cell_labels[iteration - n]) < convergence_rate)
Stop rules
Yes
return cell_labels
BREAK
ENDIF
ENDWHILE
Final model
END
