# Supplementary material for: SCINA: A Semi-Supervised Subtyping Algorithm of Single Cells and Bulk Samples
Source: Genes (Basel). 2019 Jul 12;10(7):531. doi: 10.3390/genes10070531 (PMC6678337; doi:10.3390/genes10070531)
Supplement: Supplementary file 1 [file genes-10-00531-s001.zip › Supplementary Materials/Figure S3.pdf]

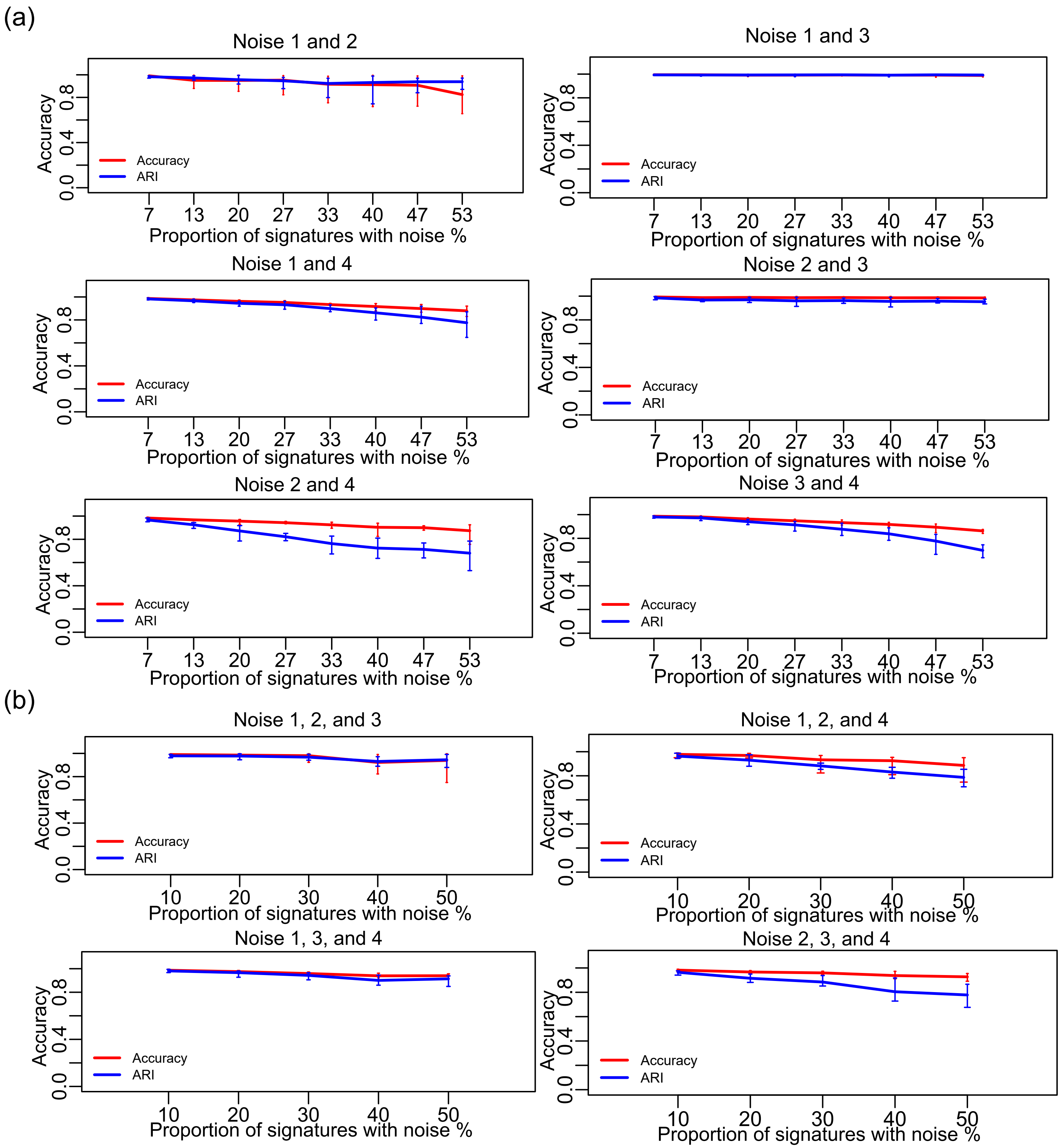
(a)
Noise 1 and 2
Noise 1 and 3
Noise 1 and 4
Noise 2 and 3
Noise 2 and 4
Noise 3 and 4
Accuracy
ARI
0.0 0.4 0.8
7 13 20 27 33 40 47 53
Proportion of signatures with noise %
(b)
Noise 1, 2, and 3
Noise 1, 2, and 4
Noise 1, 3, and 4
Noise 2, 3, and 4
Accuracy
ARI
0.0 0.4 0.8
10 20 30 40 50
Proportion of signatures with noise %
