# Supplementary figures and images for: SCINA: A Semi-Supervised Subtyping Algorithm of Single Cells and Bulk Samples
Source: Genes (Basel). 2019 Jul 12;10(7):531. doi: 10.3390/genes10070531 (PMC6678337; doi:10.3390/genes10070531)

## K-means

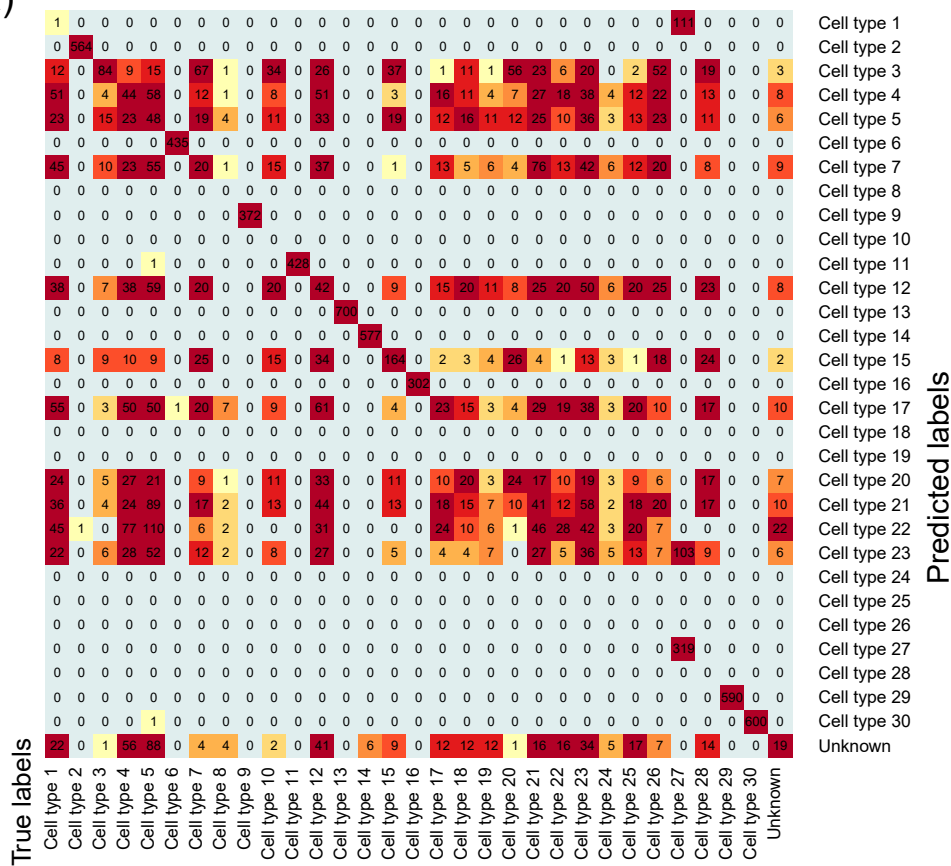

(b)

Seurat

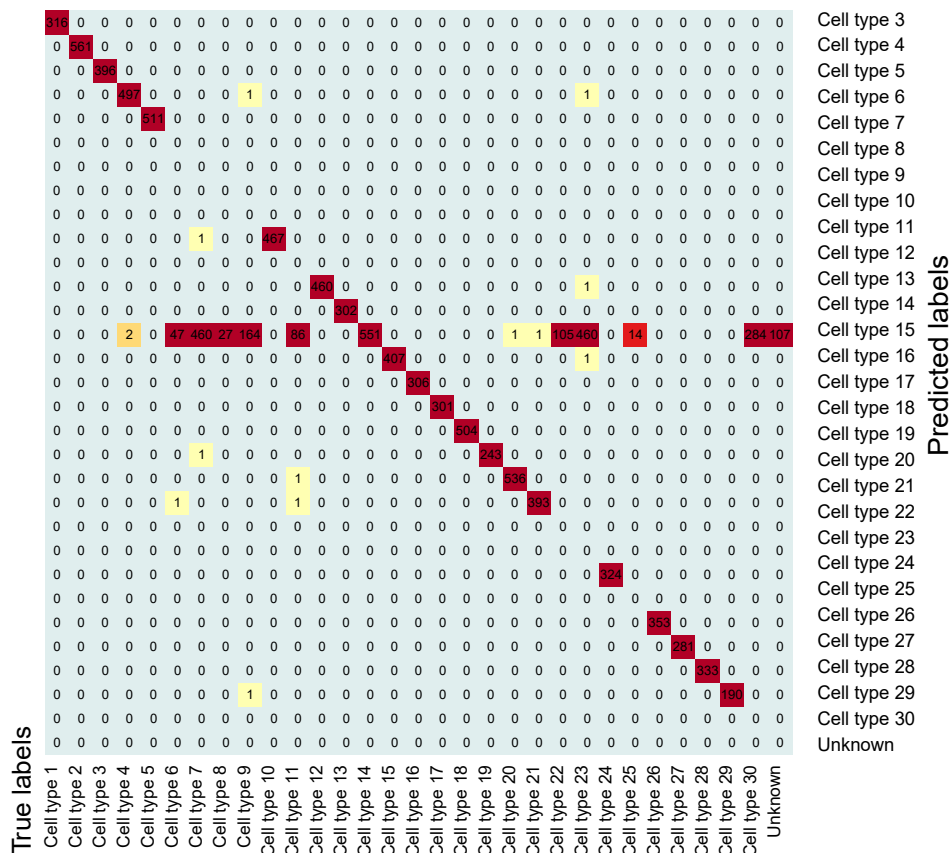

Supplement: Supplementary file 1 [file genes-10-00531-s001.zip › Supplementary Materials/Figure S2.pdf]

(a)

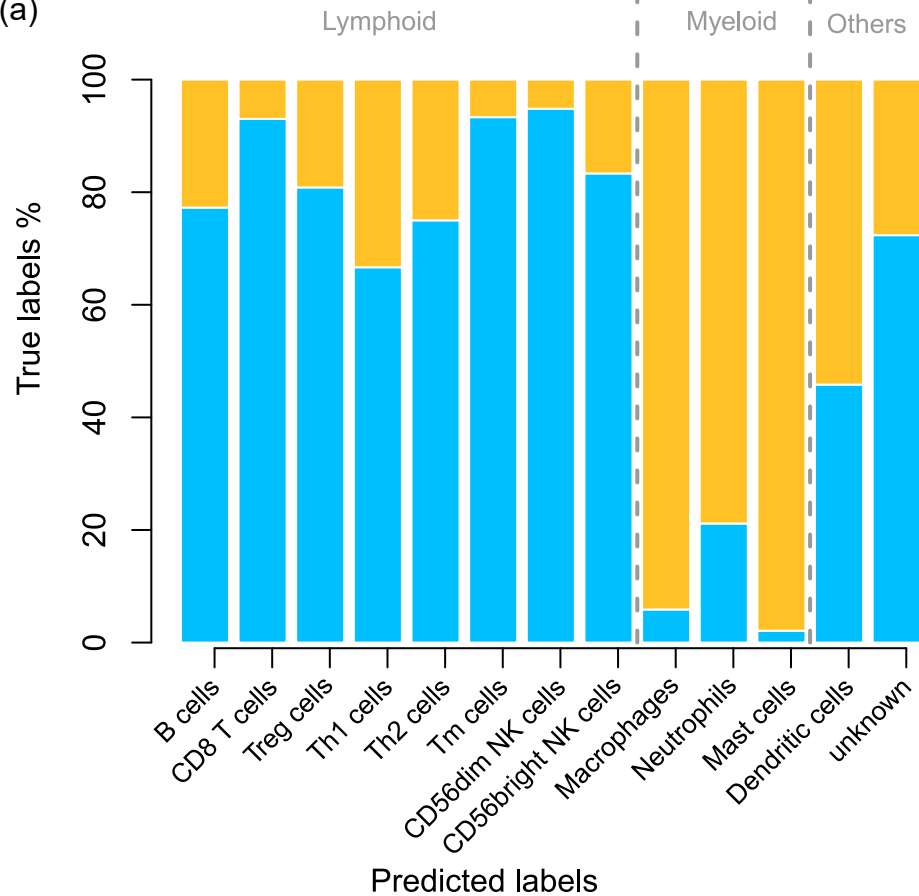

(b)

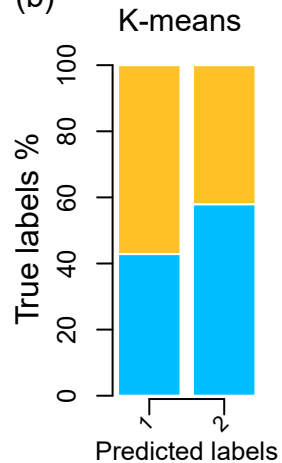

(c)

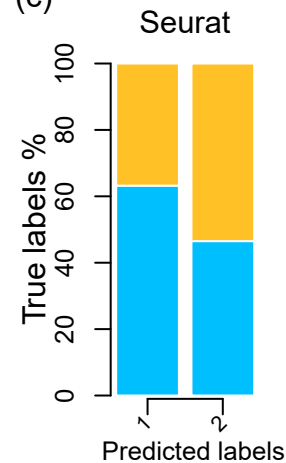

(d)

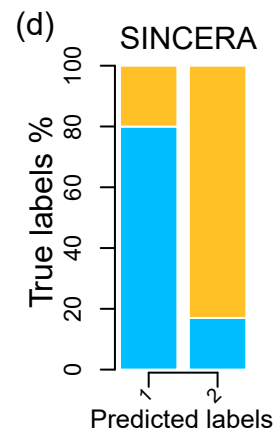

(e)

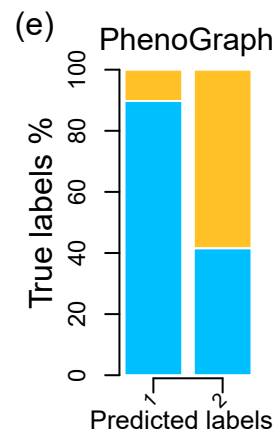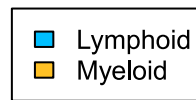

Supplement: Supplementary file 1 [file genes-10-00531-s001.zip › Supplementary Materials/Figure S6.pdf]

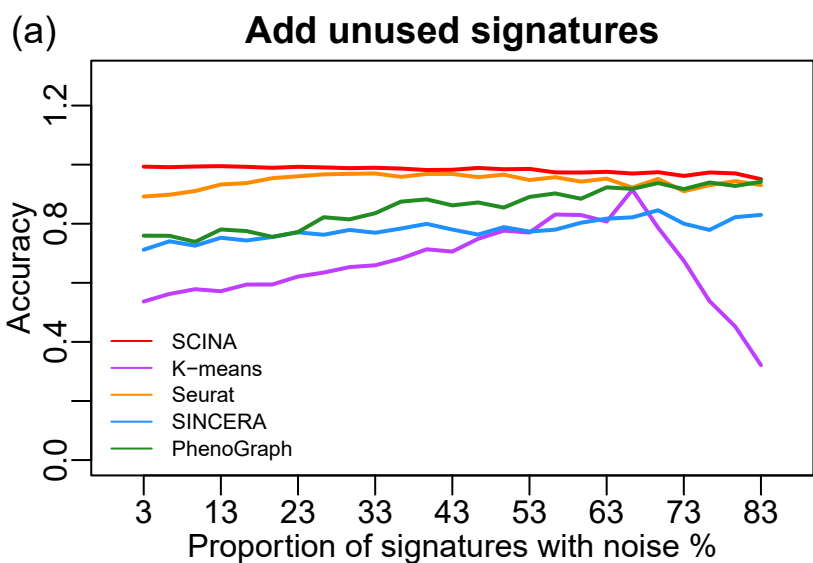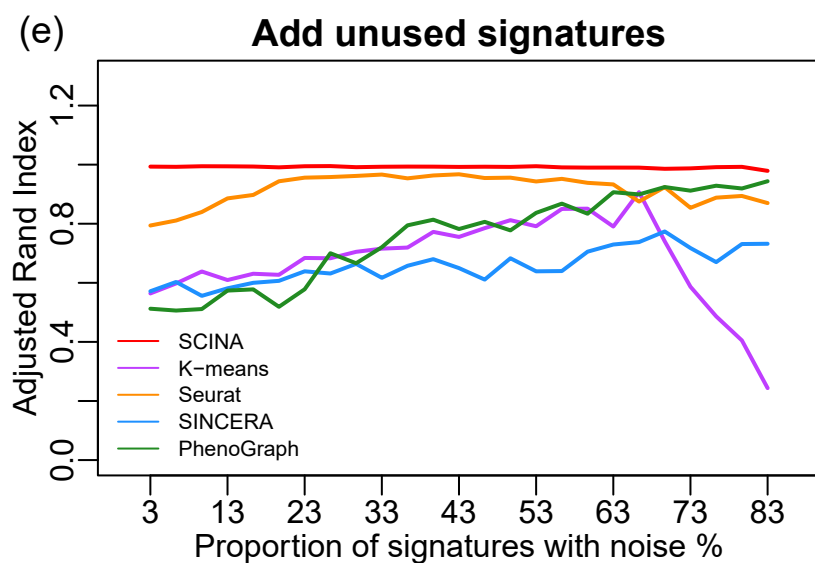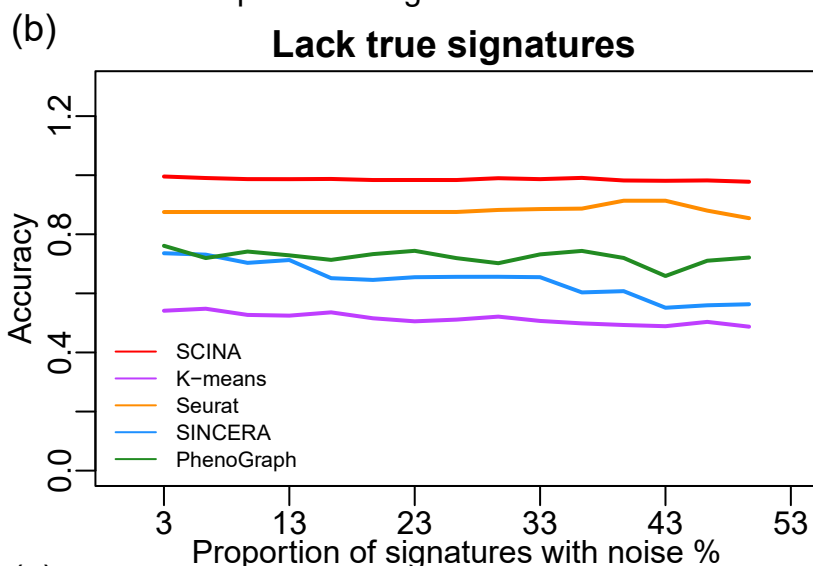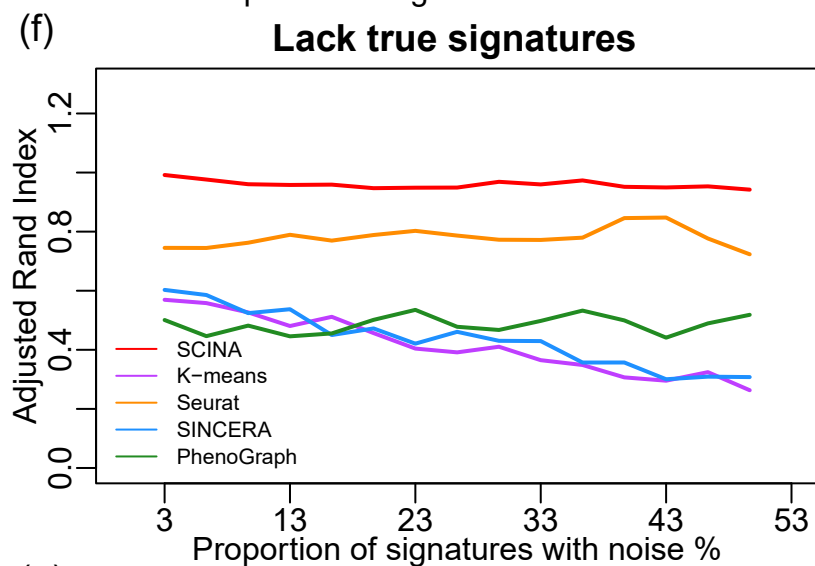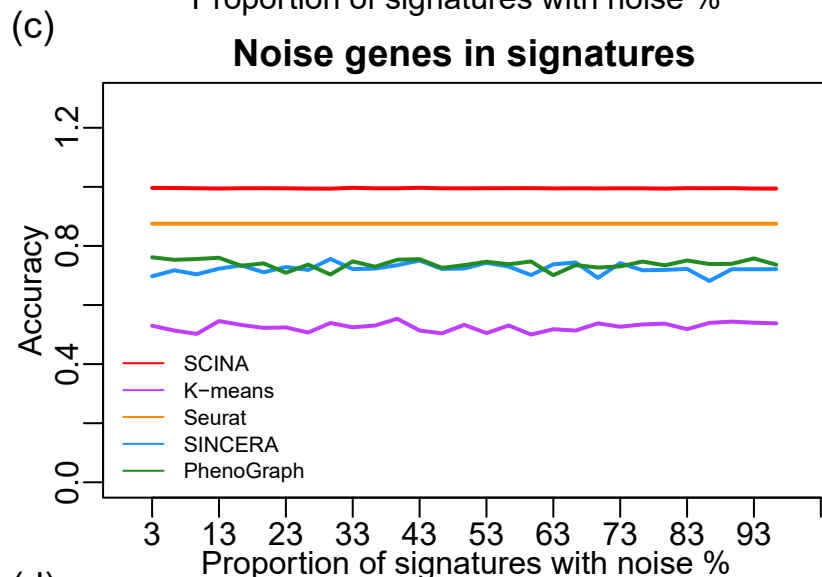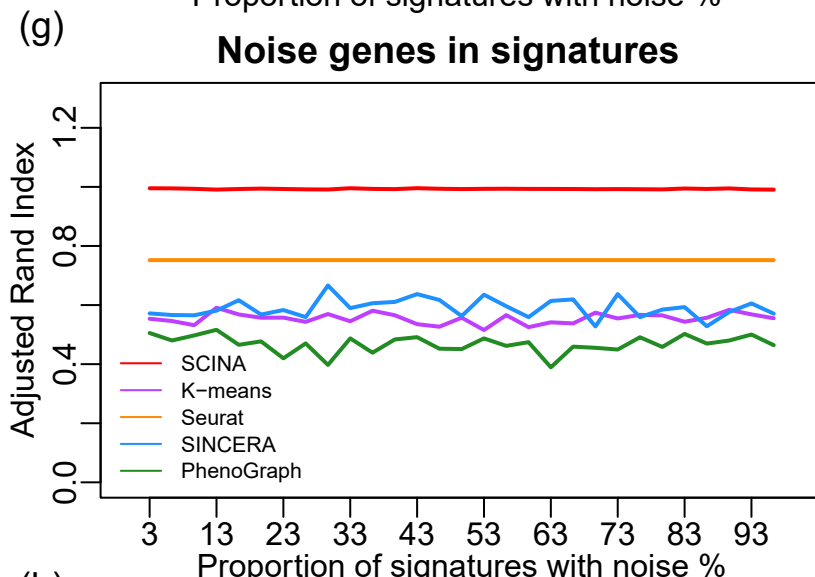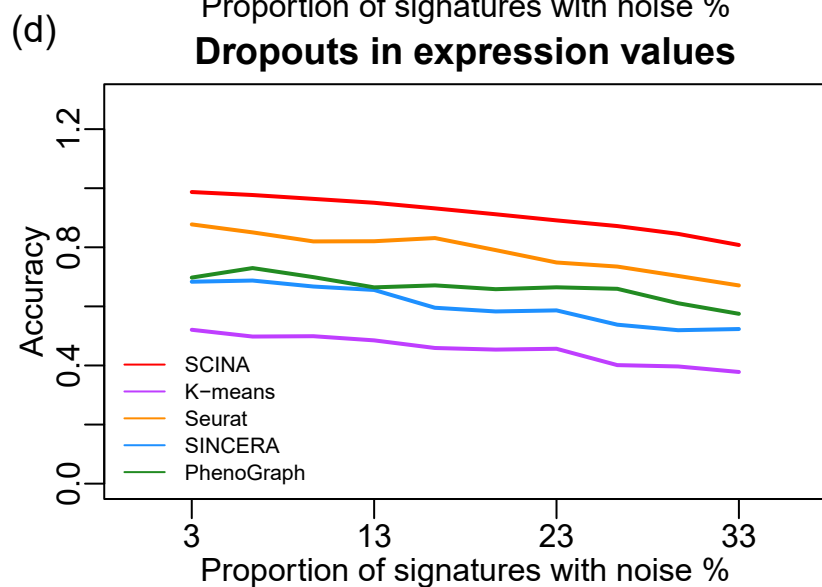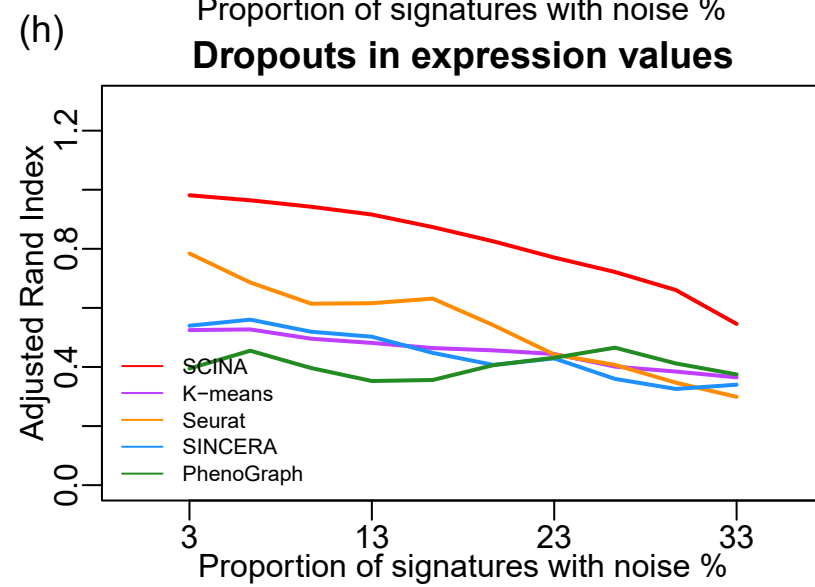

Supplement: Supplementary file 1 [file genes-10-00531-s001.zip › Supplementary Materials/Figure S4.pdf]

(a)

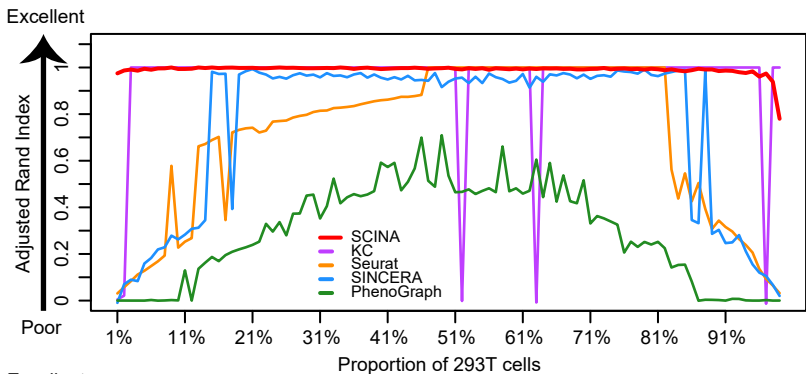

(b)

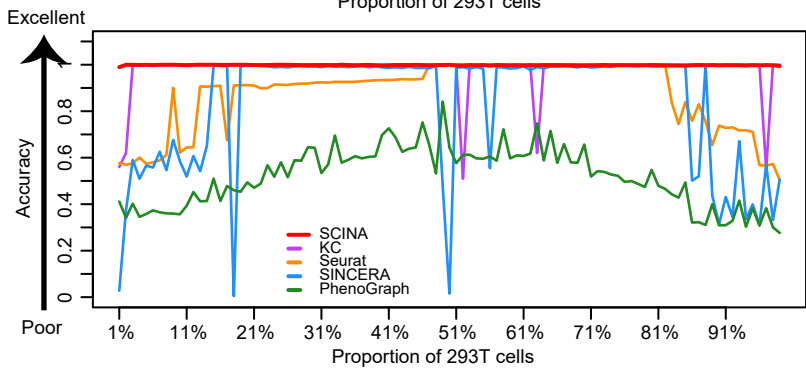

(c)

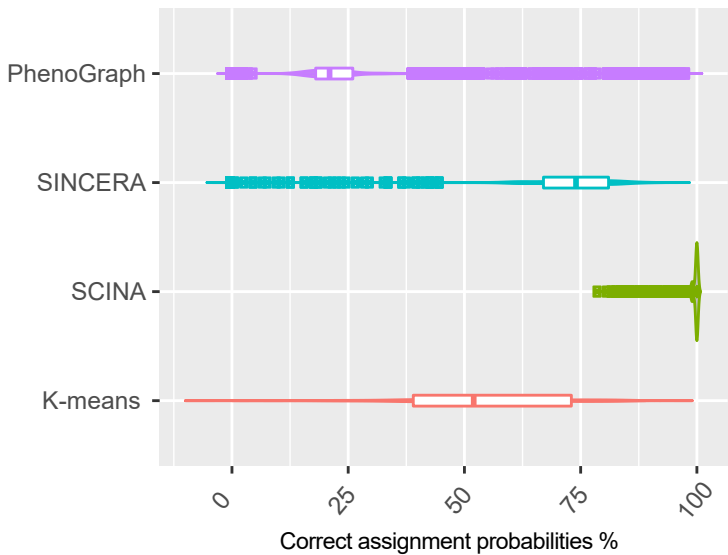

Supplement: Supplementary file 1 [file genes-10-00531-s001.zip › Supplementary Materials/Figure S5.pdf]
